# Identify glioma recurrence and treatment effects with triple-tracer PET/CT

**DOI:** 10.1186/s12880-021-00624-1

**Published:** 2021-05-31

**Authors:** Cong Li, Chang Yi, Yingshen Chen, Shaoyan Xi, Chengcheng Guo, Qunying Yang, Jian Wang, Ke Sai, Ji Zhang, Chao Ke, Fanfan Chen, Yanchun Lv, Xiangsong Zhang, Zhongping Chen

**Affiliations:** 1 Sun Yat-Sen University Cancer Center, State Key Laboratory of Oncology in South China, Collaborative Innovation Center for Cancer Medicine, Guangzhou, 510060 China; 2 Department of Nuclear Medicine, The First Affiliated Hospital of Sun Yat-Sen University, Guangzhou, 510080 China; 3 Department of Neurosurgery, The First Affiliated Hospital of Shenzhen University/Shenzhen Second People’s Hospital, Shenzhen, 518035 China

**Keywords:** ^18^F-FDOPA, ^13^N-NH_3_, ^18^F-FDG, Glioma recurrence, Treatment effects

## Abstract

**Background:**

Differential diagnosis of tumour recurrence (TuR) from treatment effects (TrE), mostly induced by radiotherapy and chemotherapy, is still difficult by using conventional computed tomography (CT) or magnetic resonance (MR) imaging. We have investigated the diagnostic performance of PET/CT with 3 tracers, ^13^N-NH_3_, ^18^F-FDOPA, and ^18^F-FDG, to identify TuR and TrE in glioma patients following treatment.

**Methods:**

Forty-three patients with MR-suspected recurrent glioma were included. The maximum and mean standardized uptake values (SUVmax and SUVmean) of the lesion and the lesion-to-normal grey-matter cortex uptake (L/G) ratio were obtained from each tracer PET/CT. TuR or TrE was determined by histopathology or clinical MR follow-up for at least 6 months.

**Results:**

In this cohort, 34 patients were confirmed to have TuR, and 9 patients met the diagnostic standard of TrE. The SUVmax and SUVmean of ^13^N-NH_3_ and ^18^F-FDOPA PET/CT at TuR lesions were significantly higher compared with normal brain tissue (^13^N-NH_3_ 0.696 ± 0.558, 0.625 ± 0.507 vs 0.486 ± 0.413; ^18^F-FDOPA 0.455 ± 0.518, 0.415 ± 0.477 vs 0.194 ± 0.203; both *P* < 0.01), but there was no significant difference in ^18^F-FDG (6.918 ± 3.190, 6.016 ± 2.807 vs 6.356 ± 3.104, *P* = 0.290 and 0.493). L/G ratios of ^13^N-NH_3_ and ^18^F-FDOPA were significantly higher in TuR than in TrE group (^13^N-NH_3,_ 1.573 ± 0.099 vs 1.025 ± 0.128, *P* = 0.008; ^18^F-FDOPA, 2.729 ± 0.131 vs 1.514 ± 0.141, *P* < 0.001). The sensitivity, specificity and AUC (area under the curve) by ROC (receiver operating characteristic) analysis were 57.7%, 100% and 0.803, for ^13^N-NH_3_; 84.6%, 100% and 0.938, for ^18^F-FDOPA; and 80.8%, 100%, and 0.952, for the combination, respectively.

**Conclusion:**

Our results suggest that although multiple tracer PET/CT may improve differential diagnosis efficacy, for glioma TuR from TrE, ^18^F-FDOPA PET-CT is the most reliable. The combination of ^18^F-FDOPA and ^13^N-NH_3_ does not increase the diagnostic efficiency, while ^18^F-FDG is not worthy for differential diagnosis of glioma TuR and TrE.

## Introduction

Glioma is the most common primary brain tumours, and the prognosis of patients with high-grade glioma is very poor [1]. Despite first-line treatments, including maximal safe surgical resection, irradiation, and adjuvant chemotherapy, the median survival time for most malignant glioblastoma patients is still only 12–15 months [2]. Recurrence is a critical clinical problem in glioma patients, but the diagnosis of recurrent glioma is difficult using computed tomography (CT) or magnetic resonance (MR) imaging because radiotherapy and chemotherapy are associated with a high incidence of treatment-related imaging changes termed treatment effects (TrE), which is occasionally referred to as pseudoprogression and radiation necrosis. This phenomenon is believed to be related to the destruction of the blood–brain barrier after radiation therapy and chemotherapy, leading to increased leakiness of contrast agent exhibiting enhancement in the area on MR imaging, which is similar to the imaging findings of tumour recurrence (TuR) [3]. Although advanced multimodal MR imaging plays an important role in the post-treatment follow-up of glioma patient care in recent years, the differential diagnosis in clinical image evaluation between TrE and TuR in glioma patients remains challenging because they have overlapping clinical and radiologic features [4–6]. The diagnosis of TuR requires a change in the therapeutic approach to a more active anti-tumour strategy, whereas a diagnosis of TrE supports the effectiveness of current treatment. Therefore, accurate and reliable imaging evaluation is very important for making clinical decisions.

Positron emission tomography/computed tomography (PET/CT) is a molecular imaging technique allowing in vivo quantitative measurement of biological processes noninvasively, which has become an integral supplemental imaging tool for differential diagnosis of brain lesions beyond MR [7]. Some PET tracers, such as ^18^F-fluorodeoxyglucose (^18^F-FDG), ^13^N-ammonia (^13^N-NH_3_), ^11^C-methylmethionine (^11^C-MET), ^18^F-fluoroethyl-L-tyrosine (^18^F-FET), and ^18^F-fluoro-L-dihydroxy-phenylalanine (^18^F-FDOPA), have been used for imaging gliomas [7–10]. The Response Assessment in Neuro-Oncology working group and European Association for Neuro-Oncology have also recommended the clinical use of PET/CT imaging in gliomas, and they emphasize that PET/CT exhibits increased diagnostic accuracy than MR when differentiating TuR from TrE [11]. However, no standard imaging technology is currently available to achieve a differential diagnosis, and few articles compare different metabolic types of PET/CT tracers used in this critical issue of the glioma follow-up process. This study aims to explore the clinical potential of ^13^N-NH_3_, ^18^F-FDOPA, and ^18^F-FDG PET/CT, which represent three different types of tracers in the differential diagnosis of TuR and TrE in glioma patients, to reduce clinical misdiagnosis.

## Materials and methods

### Patients

Patients treated at our hospital between September 2016 and December 2019 with suspicion of recurrent gliomas based on MR imaging demonstrating new enhancement lesions and the desire for PET/CT examination were included in this study. The initial diagnosis for these patients with histopathologic glioma according to revised 2016 World Health Organization (WHO) criteria, and TuR or TrE diagnosis was determined by histology if the patient received repeated surgical resection or standard clinical MR follow-up for at least 6 months at 2- to 3-month intervals after the PET/CT exam. This study was approved by the ethics committee of our cancer centre, and informed consent was obtained from all individual participants included in the study.

Forty-three patients (16 females and 27 males) were enrolled in the study. The average age was 41.74 ± 12.71 years (range, 14–65 years). Thirty-four patients received all three tracer examinations. Three patients only underwent ^13^N-NH_3_ and ^18^F-FDG PET/CT, and six patients only underwent ^18^F-FDOPA PET/CT due to the patient's desire or shortage of tracers. Detailed patient characteristics of each tracer are listed in Table 1. Except for one patient’s lesion located in the left cerebellum, the other patients’ lesions were mainly located in the temporal lobe, frontal lobe or parietal lobe, including 23 cases on the right side and 19 cases on the left side. All patients received radiotherapy before, and the mean dose was 59.23 ± 1.84 Gy (range, 54–66 Gy). Only one patient did not receive adjuvant chemotherapy and the others received temozolomide-based adjuvant chemotherapy. The median interval time from primary diagnosis to PET/CT examination was 19.10 months (range, 4.10–88.20 months). The median interval between radiotherapy and PET/CT was 15.90 months (range, 1.67–84.77 months).

**Table 1:** Patient characteristics of each PET/CT tracer

Characteristics		PET		
	N	NH3	DOPA	FDG
N	43	34	43	37
*Gender*				
Male	27	20	27	23
Female	16	14	16	14
Age (years)	38.5 ± 6.37	40.38 ± 12.41	38.5 ± 6.37	40.28 ± 6.36
*WHO grade*				
II	12	11	12	12
III	15	10	15	11
IV	16	13	16	14
*IDH1*				
Mutation	24	21	24	21
Wide type	13	7	13	10
NA	6	6	6	6
*Final diagnosis*				
TuR	34	26	34	29
TrE	9	8	9	8

*WHO* World Health Organization, *NA* not achieved, *TuR* tumour recurrence, *TrE* treatment effects

### PET/CT imaging protocol

Tracers were synthesized through a commercially available system for isotope generation (Cyclone-10, Ion Beam Applications S.A., Belgium; AllinOne synthesizer, Trasis, Belgium). ^13^N-NH_3_ and ^18^F-FDG were produced as previously mentioned [7]. ^18^F-FDOPA was produced using the method of Libert et al. [12]. The radio-chemical purity of ^13^N-NH_3_ was > 99%, ^18^F-FDOPA was > 98%, and ^18^F-FDG was > 95%. PET/CT examinations were performed on a Gemini GXL 16 scanner (Philips, Netherlands) in 3-dimensional acquisition mode. The reconstruction protocols of PET/CT have been published in detail previously [7]. Briefly, patients underwent a fast for at least 4 h before the ^18^F-FDOPA examination. Ten minutes after the intravenous injection of 2 MBq/kg of ^18^F-FDOPA, a dedicated CT scan of the brain (120 kV, 80 mAs, 3 mm slice collimation) was performed followed by a brain-centred static 3D PET acquisition of 10 min. Then, a clear PET image was obtained. ^18^F-FDG PET/CT was performed after ^13^N-NH_3_ with a minimum interval of 2 h on another day within 1 week, and patients remained untreated until the PET/CT study was completed. All patients had fasted for at least 6 h before ^13^N-NH_3_ and ^18^F-FDG examination. First, after intravenous injection of 7.4 MBq (0.20 mCi)/kg of ^13^N-NH_3_, patients rested in a quiet room, and PET/CT was performed 10 min later. Then, a dose of 5.18 MBq (0.14 mCi)/kg ^18^F-FDG was injected intravenously, and serial scanning was performed approximately 30–45 min after the injection with the patient resting with their eyes closed. Finally, PET/CT images were reconstructed using the LOR-RAMLA algorithm with low-dose CT images for attenuation correction.

### PET image parameters

PET/CT images were evaluated by two experienced nuclear medicine physicians independently who were blinded to the final clinical diagnosis of the lesions. The tracers’ uptake of the lesion was evaluated by quantitative analysis. A region of interest (ROI) was placed over the entire lesion on the transverse PET/CT image by the nuclear physicians. For lesions with reduced or equal uptake, the ROI was drawn based on the anatomical information on the brain lesions presented by previous MR. The grey-matter ROI was drawn in the lobe contralateral to the lesion according to the method we reported before [13, 14]. After reaching the consensus of the target lesions and ROI, the maximum and mean standardized uptake value (SUV) of the lesion was detected as SUVmax and SUVmean. The lesion-to-normal grey-matter cortex uptake (L/G) ratio was calculated as the SUVmax of the lesion divided by the average SUV of the contralateral normal grey-matter (CNGM) cortex.

### True diagnosis of lesion

TuR or TrE was determined by histopathology if repeated surgical resection was performed. When the histopathologic examination was not available, clinical follow-up, including longitudinal MR and clinical symptoms, was considered the standard of truth according to the Response Assessment in Neuro-Oncology (RANO) criteria [15] and our previous report [4, 16]. Briefly, the lesion was defined as TuR based on a progressive increase in size and/or the number of enhancing lesions with the development of neurologic symptoms. TrE was defined based on a reduced or stable lesion size on subsequent follow-up images with a stable or improved clinical condition for at least 6 months without additional steroid treatment.

### Statistical analysis

All statistical analyses were performed using IBM SPSS Statistics 23.0 (SPSS Inc. Chicago, IL, USA) software. Continuous variables are expressed as the mean ± standard deviation (SD) or medians with range. Student’s t-test or Chi-square tests were used to compare the differences between the TuR and TrE groups. A paired Student’s t-test was used to compare lesions and CNGM within each PET/CT tracer. The overall survival of glioma patients after PET/CT examination was evaluated using the log rank test with Kaplan–Meier analysis. The receiver operating characteristic (ROC) curve was chosen to assess the performance of PET/CT parameters in differentiating between TuR and TrE. The optimal cutoff values were determined by maximizing the Youden’s index value. *P* < 0.05 was considered statistically significant.

## Results

### Tumour recurrence (TuR) and treatment effects (TrE)

**Fig. 1:**
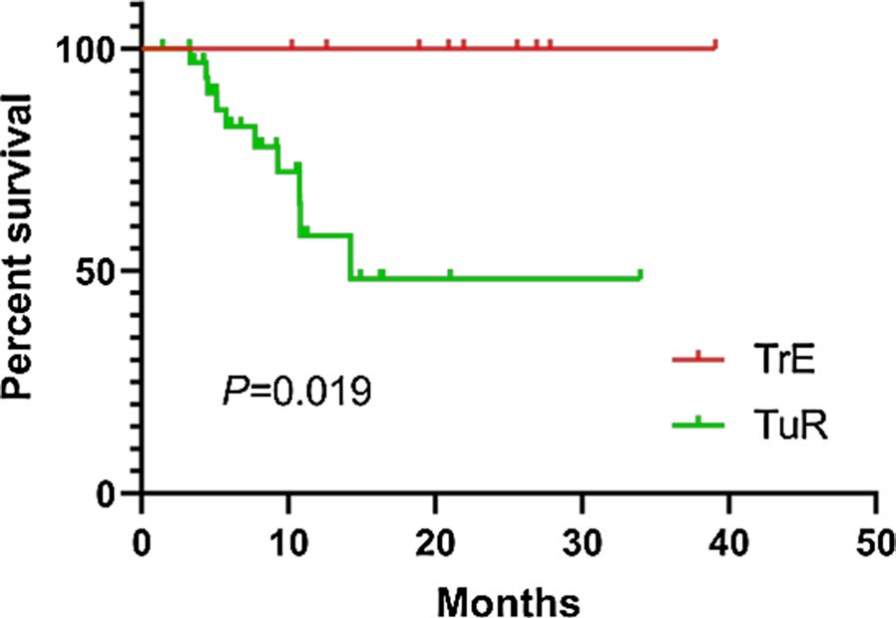
The overall survival curves of glioma patients after PET/CT examination according to final diagnosis with TrE and TuR

Finally, thirty-four patients were diagnosed with TuR, whereas the remaining 9 were diagnosed with TrE. Note that seven patients were diagnosed from histopathology after reoperation, and the remaining patients were diagnosed based on clinical and radiological follow-up for more than 6 months. TrE occurred more frequently in females (7/16, 43.75%) compared with males (2/27, 7.41%) (*P* = 0.02). Among the 37 patients with IDH1 mutation status results, the probability of IDH1 mutation in the TrE group was 100% (8/8), whereas that in the TuR group was 55.17% (16/29) (*P* = 0.03). The two groups exhibited no significant differences in terms of patients’ age (*P* = 0.54), WHO grade (*P* = 0.09), radiotherapy dose (*P* = 0.37) or the time interval between radiotherapy and diagnosis surgery (*P* = 0.36 and 0.39) (Table 2). The median follow-up time from PET/CT performed was 10.23 months (95% CI, 9.26–14.63 months). Kaplan–Meier analysis showed that patients diagnosed with TrE exhibited improved survival compared with those diagnosed with TuR (log-rank test, x^2^ = 5.524, *P* = 0.019) (Fig. 1).

**Table 2:** Patient characteristics of TuR and TrE

Characteristics	TuR	TrE		*P* values
N	34	9		
*Gender*				
Male	25	2	X^2^ = 8.02	0.02^*^
Female	9	7		
Age (years)	41.12 ± 12.52	44.11 ± 13.91	t = 0.62	0.54
*WHO grade*				
II	10	2		
III	10	6	X^2^ = 4.63	0.09
IV	14	1		
*IDH1*				
Mutation	16	8	X^2^ = 5.53	0.03^*^
Wide type	13	0		
Dose of RT (Gy)	59.41 ± 1.81	58.78 ± 1.99	t = -0.92	0.37
interval from RT (Mon.)	22.51 ± 21.05	29.61 ± 16.61	t = 0.93	0.36
interval from First Diagnosis (Mon.)	25.29 ± 21.28	32.03 ± 16.57	t = 0.88	0.39

*WHO* World Health Organization; *Mon*. month, *TuR* tumour recurrence, *TrE* treatment effects, *RT* radiotherapy

### PET/CT findings

**Fig. 2:**
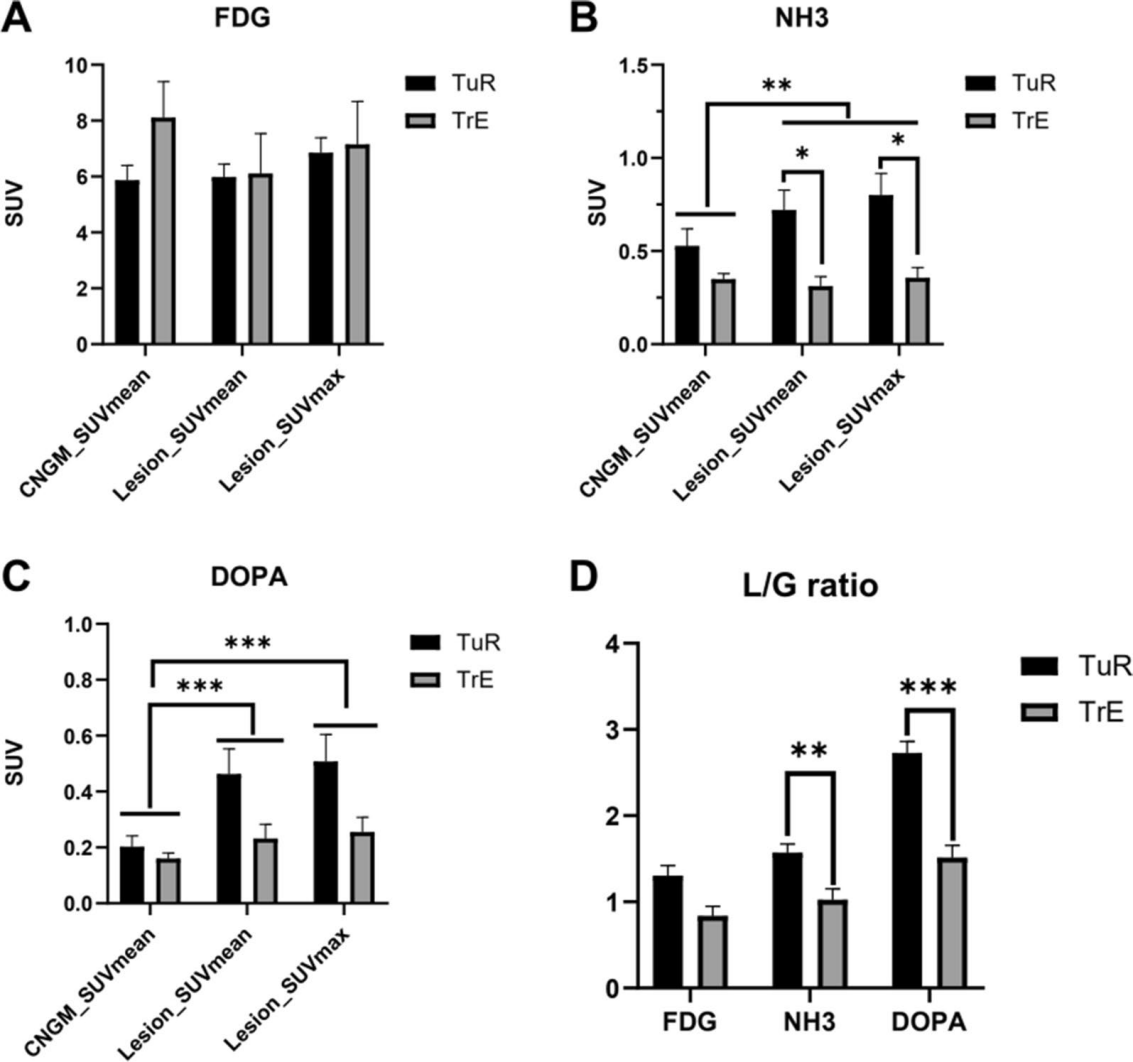
The SUVmax and SUVmean of the lesions compared with SUVmean of CNGM and with groups of TuR and TrE in different PET tracers (**a** ^18^F-FDG; **b** ^13^N-NH_3_; **c** ^18^F-FDOPA). The L/G ratios of ^18^F-FDG, ^13^N-NH_3_ and ^18^F-FDOPA were compared in the TuR and TrE groups (**d**). *P < 0.05, **P < 0.01, and ***P < 0.001

The SUVmax and SUVmean of lesions were significantly increased compared with the SUVmean of contralateral normal grey matter (CNGM) in ^13^N-NH_3_ and ^18^F-FDOPA PET (^13^N-NH_3_ 0.696 ± 0.558, 0.625 ± 0.507 vs 0.486 ± 0.413; ^18^F-FDOPA 0.455 ± 0.518, 0.415 ± 0.477 vs 0.194 ± 0.203; both *P* < 0.01), but there was no significant difference in ^18^F-FDG (6.918 ± 3.190, 6.016 ± 2.807 vs 6.356 ± 3.104, *P* = 0.290 and 0.493) (Fig. 2a–c).

**Fig. 3:**
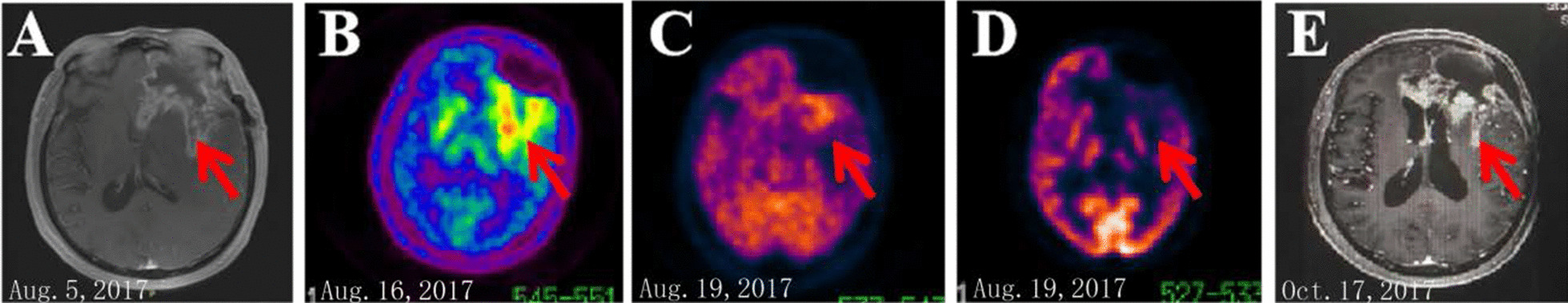
A representative patient with glioma TuR. Contrast MR image (**a**) reveals an enhanced lesion with obviously increased uptake of ^18^F-FDOPA (**b**), slightly increased uptake of ^13^N-NH_3_ (**c**), and decreased uptake of ^18^F-FDG (**d**). Two months later, contrast MR showed that the lesions continued to increase (**e**), and the patient died 8 months later

**Fig. 4:**
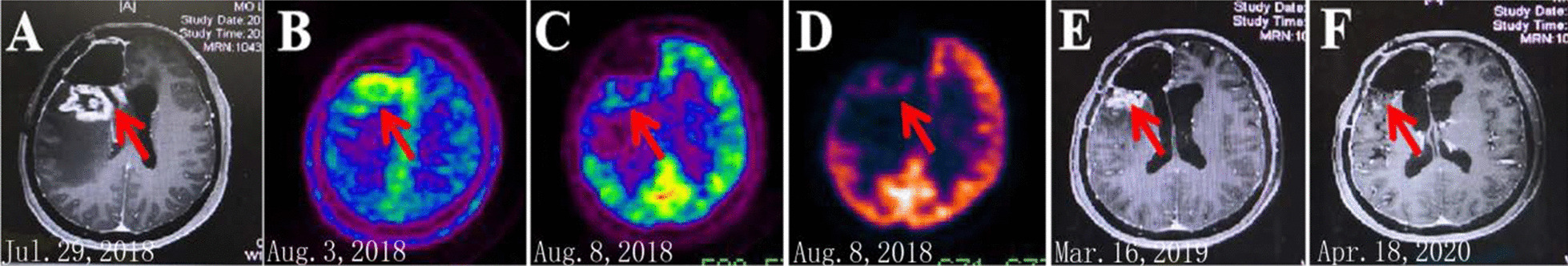
A representative patient with TrE. Contrast MR image (**a**) presents an enhanced lesion with slightly increased uptake of ^18^F-FDOPA (**b**) and no increased uptake of ^13^N-NH_3_ (**c**) and ^18^F-FDG (**d**). Follow-up MR revealed that the enhanced lesions gradually decreased and disappeared (**e**, **f**)

The lesions’ SUVmax and SUVmean of ^13^N-NH_3_ were significantly different in patients with TuR compared to patients with TrE (Fig. 2b), whereas TuR exhibited significantly increased L/G ratios than TrE both in ^13^N-NH_3_ and ^18^F-FDOPA PET/CT (^13^N-NH_3_, 1.573 ± 0.099 vs 1.025 ± 0.128, *P* = 0.008; ^18^F-FDOPA, 2.729 ± 0.131 vs 1.514 ± 0.141, *P* < 0.001) but not in ^18^F-FDG (Fig. 2d). Figures 3 and 4 report different PET/CT performances in two typical cases of TuR and TrE.

**Fig. 5:**
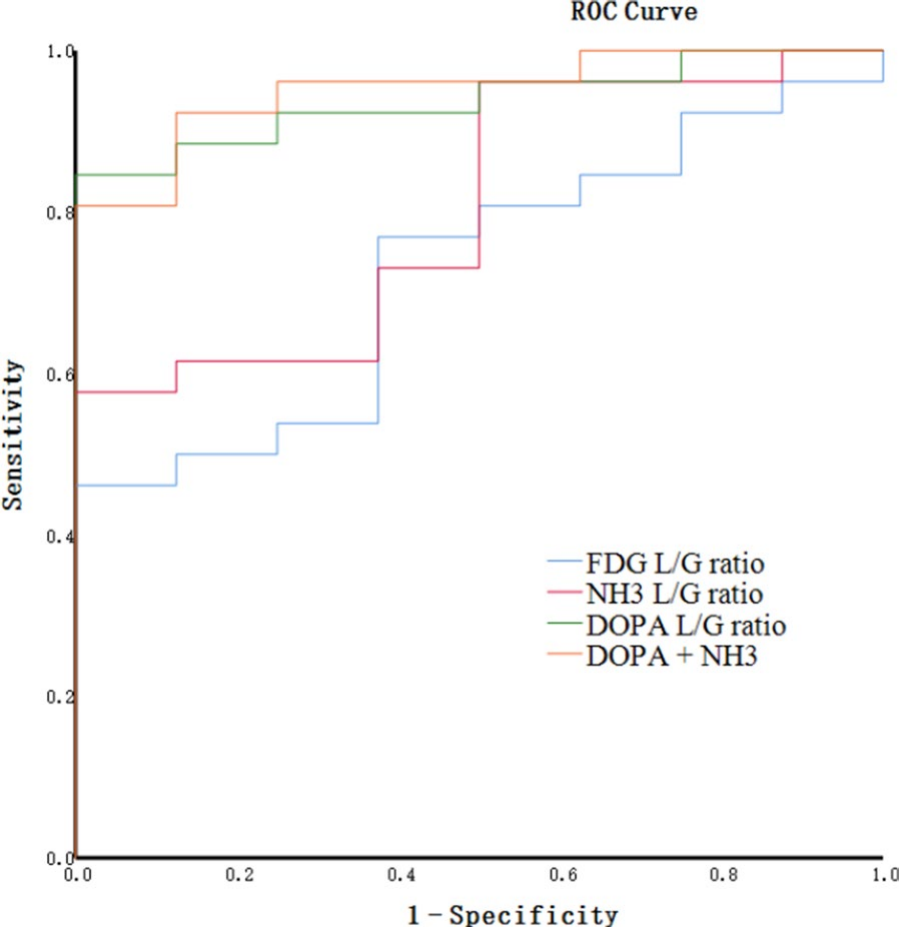
ROC curve analysis of the L/G ratio to differentiate TuR and TrE. The area under the curve (AUC) was 0.726 for ^18^F-FDG, 0.803 for ^13^N-NH_3_, 0.938 for ^18^F-FDOPA and 0.952 for the combination of ^18^F-FDOPA and ^13^N-NH_3_

ROC analysis for differentiation between TuR and TrE yielded an optimal L/G ratio of 2.165 for ^18^F-FDOPA (sensitivity, 84.6%; specificity, 100%; AUC, 0.938; 95% CI [0.859–1.000];), 1.260 for ^18^F-FDG (sensitivity, 46.2%; specificity, 100%; AUC, 0.726; 95% CI [0.547–0.905];), and 1.515 for ^13^N-NH_3_ (sensitivity, 57.7%; specificity, 100%; AUC, 0.726; 95% CI [0.547–0.905]) (Table 3). The L/G ratio of ^18^F-FDOPA exhibited the best PET/CT parameters compared to ^18^F-FDG and ^13^N-NH_3_. The area under the ROC curves of the L/G ratio evaluation of ^13^N-NH_3_ and ^18^F-FDOPA was 0.803 and 0.938, respectively (Fig. 5). Combining the L/G ratio evaluation of ^18^F-FDOPA and ^13^N-NH_3_, the diagnostic performance did not show further improvement, yielding a sensitivity of 80.8%, a specificity of 100%, and an AUC of 0.952.

**Table 3:** ROC curve analyses of the L/G ratio of FDG, NH3, DOPA, and the combination of NH3 and DOPA in differentiating TuR and TrE

PET index	AUC	SE of AUC	*P* values	95% CI				
				lower	upper	Optimal cutoff	Specificity%	Sensitivity%
FDG	.726	.091	.056	.547	.905	1.260	100	46.2
NH3	.803	.081	.011	.644	.962	1.515	100	57.7
DOPA	.938	.040	.000	.859	1.000	2.165	100	84.6
DOPA + NH3	.952	.035	.000	.883	1.000	0.832	100	80.8

*AUC* area under curve, *SE* standard error, *TuR* tumour recurrence, *TrE* treatment effects

## Discussion

The prognosis of patients with gliomas is very poor once recurrence occurs after comprehensive treatment. Some studies have demonstrated that the median survival time of glioma patients is only 9–10 months after the first tumour recurrence [17, 18]. Generally, the follow-up of glioma patients after or during comprehensive treatment mainly depends on the MR examination. However, TrE may lead to mimicking of tumour recurrence because they exhibit similar features on contrast-enhanced MR, which are also known as early effects (i.e., pseudoprogression) or late effects, such as radiation necrosis mainly based on timing. Although pseudoprogression and radiation necrosis are thought to represent distinct clinical and pathophysiologic mechanisms, they share many histologic similarities, such as inflammatory infiltrates and necrosis, which translate into similar imaging characteristics [19]. This similarity makes it difficult to identify TuR and TrE with the most commonly used MR images, especially contrast-enhanced MR. Although several advanced MR techniques, such as amide proton transfer, diffusion, perfusion, and spectroscopy imaging, can improve the differential diagnosis and are widely used in clinical applications [4, 20], their accuracy and efficacy remain limited. The final diagnosis requires longitudinal MR observation for several months or repeat surgery. This process is resource-intensive as well as burdensome for the patients, and the longitudinal MR observation also delays definitive treatment. Then, experienced doctors will use PET/CT, which can reflect the metabolism of lesions, to assist MR for optimal diagnosis.

In this study, we investigated the diagnostic performance of multitracer PET/CT techniques, including ^13^N-NH_3_, ^18^F-FDOPA, and ^18^F-FDG, in distinguishing TuR from TrE in a cohort of patients with suspected recurrent gliomas. ^18^F-FDG is the most widely used tracer in PET/CT imaging and reflects the glucose metabolism level of tumours, especially malignant tumours. However, ^18^F-FDG may have some limitations in differentiating intracranial tumours with low metabolic levels from normal brain tissues, inflammation, and benign tumours because it accumulates largely in normal brain tissue, leading to small differences between glioma lesions and normal brain tissue [21]. Therefore, ^18^F-FDG is not very good for glioma display, especially for low-grade glioma and lesions close to grey matter. This limitation was also confirmed in this study, in which ^18^F-FDG metabolism was not significantly different between lesions and contralateral grey cortical tissue. The low L/G ratio also limits its role in the differential diagnosis of TuR and TrE, and no significant difference was found in this study.

^13^N-NH_3_ is another PET imaging agent that is low fat-soluble and has a small diameter, allowing it to penetrate the blood–brain barrier (BBB). We have previously reported that the uptake of ^13^N-NH_3_ is superior to ^18^F-FDG not only in enabling differentiation between glioma and non-neoplastic lesions but also in separating low-grade gliomas (LGG) from high-grade gliomas [13, 14]. This difference may be due to the uptake of ^13^N-NH_3_ in normal brain tissue being relatively lower than ^18^F-FDG, whereas the uptake of ^13^N-NH_3_ in glioma is significantly increased. Therefore, the L/G ratio of ^13^N-NH_3_ PET/CT imaging is higher in tumourous lesions, making it more beneficial to distinguish glioma from some inflammatory or benign tumours. Furthermore, our other study [9] also found that the increased ^13^N-NH_3_ uptake in recurrent glioma and the absent or lower uptake in radiation necrosis due to perfusion and glutamine synthetase activity in the recurrent tumour is higher than that in the TrE. In this study, we also found that ^13^N-NH_3_ is a promising tracer for separating TuR from TrE. In addition, SUVmax, SUVmean, and L/G ratio all showed good performance for this purpose, and their role seemed superior to ^18^F-FDG.

Because gliomas have upregulated amino acid transporters and increased amino acid metabolism, and labelled amino acid tracers, including ^11^C-MET, ^18^F-FET, and ^18^F-FDOPA, are increasingly being widely used in gliomas in recent years with better tumour-to-background contrast for differentiation glioma grade, biopsy guiding, radiotherapy planning, therapy monitoring, and differentiation between TrE and residual or recurrent glioma [21–23]. Amino acid PET/CT typically demonstrates high uptake in glioma and low uptake in the normal brain; thus, the L/G ratio exhibits advantages over SUVmax and SUVmean of the lesion. These amino acid tracers can be used in PET/CT for suspicion of recurrent glioma based on pathophysiological differences between the actively growing tumour, which exhibits increased transport and metabolism of the amino acid; conversely, treatment-induced brain changes result in a low level of metabolism in lesions. For example, Martinez-Amador et al. [24] applied an L/CP SUVmax index to differentiate post-therapeutic changes from tumour presence with a sensitivity of 89.3%, specificity of 90.0%, positive predictive value of 96.1%, negative predictive value of 75%, and accuracy of 82.9%. Hotta Masatoshi et al. [25] found that ^11^C-MET radiomics yielded excellent outcomes for differentiating recurrent brain tumours from radiation necrosis, which outperformed the T/N ratio evaluation with areas under the curve of 0.98 and 0.73. This result means that ^11^C-MET PET/CT is useful in differentiating glioma TuR from TrE. However, another study suggests that increased uptake of ^11^C-MET, such as ^18^F-FDG, may have limited specificity in distinguishing inflammatory lesions from tumours [26]. Bashir et al. [27] found that a 20-min ^18^F-FET PET scan is a powerful tool with TBRmax (sensitivity 99%, specificity 94%) to distinguish posttreatment changes from recurrent glioblastoma 6 months postradiotherapy. Bogsrud et al. [28] reported the performance of a new type of amino acid ^18^F-fluciclovine in PET/CT of suspected residual or recurrent glioma, but the ability of ^18^F-fluciclovine PET/CT to discriminate between recurrent glioma and treatment-related changes could not be determined because no patients had confirmed treatment-related changes. More recently, ^18^F-labelled DOPA is a more widely used amino acid tracer than ^11^C-MET because it has a longer half-life of up to 110 min, whereas that of ^11^C-MET is only 20 min. Humbert et al. [8] found that ^18^F-FDOPA PET has a significant impact on the management of patients with a suspicion of brain tumour recurrence, either glioblastoma or brain metastases, but a low impact when used to evaluate residual glioblastoma infiltration after first-line radiochemotherapy or second-line bevacizumab. ^18^F-FDOPA has been increasingly used in glioma and exhibits potential value in the identification of TrE and TuR [21].

Some other tracers such as the nucleoside analog ^18^F-fluorothymidine (^18^F-FLT) and hypoxia tracer ^18^F-fluoromisonidazole (^18^F-FMISO) also displayed reliable performances in glioma imaging. ^18^F-FLT can reflect tumor proliferation rate and be a marker of glioma aggressiveness due to thymidine is a nucleoside encountered in DNA [29, 30]. But it reflects proliferative indices to variable and potentially unreliable extents [31]. Given the low accumulation of ^18^F-FLT in low-grade gliomas, ^18^F-FLT PET/CT should not be used in low-grade recurrent gliomas [32]. What’s more, Enslow et al. [33] found there was no significant difference between TrE and TuR with regard to SUVmax parameter of ^18^F-FLT PET/CT. ^18^F-FMISO is the most common radiotracer for hypoxia imaging which can distinguish glioblastomas from lower-grade gliomas due to glioblastomas presents with necrosis and hypoxic environment, whereas lower-grade gliomas do not develop necrosis, and ^18^F-FMISO PET/CT also can predict the tumor microenvironment, including necrosis, vascularization, and permeability [34]. As ischemia and hypoxia are also important mechanisms of TrE [35], ^18^F-FMISO PET/CT is limited used in differentiating TuR from TrE, but it has potential value to assess treatment response for anti-angiogenic therapy [36].

In the present study, we demonstrated the application of three typical tracers in the detection of recurrent glioma. The overall L/G ratio of ^18^F-FDOPA and ^13^N-NH_3_ is better than that of ^18^F-FDG. Further ROC analysis showed that the L/G ratio of ^18^F-FDOPA appears to outperform sensitivity to ^13^N-NH_3_ in the assessment of TrE from TuR even when the specificity is 100%. ^18^F-FDG itself has no advantage in the diagnosis of recurrent glioma, only ^13^N-NH_3_ and ^18^F-FDOPA are selected for combined diagnosis analysis. Compared with ^18^F-DOPA alone, the diagnostic sensitivity and efficiency of the combination of ^18^F-DOPA and ^13^N-NH_3_ have not been improved. These findings suggest that ^18^F-FDOPA alone may be more acceptable to patients and that the diagnostic effect is equivalent. According to our previous research, this finding may be due to the fact that ^13^N-NH_3_ PET/CT has high specificity in the diagnosis of brain tumours compared with nontumour lesions, but the sensitivity is low when differentiating these lesions from LGG, which is consistent with the research results in this paper. There may be a certain degree of similarity in metabolic level between the process of glioma recurrence after chemoradiotherapy and the formation of LGG. Thus, ^18^F-DOPA itself has high specificity (100%) and sensitivity (84.6%) in distinguishing TuR and TrE. The combination of low sensitivity ^13^N-NH_3_ PET/CT does not help.

To our knowledge, this study is the first comparison among ^13^N-NH_3_, ^18^F-FDG and ^18^F-FDOPA PET/CT in patients with suspected glioma recurrence, and ^18^F-FDOPA exhibited good performance in the differential diagnosis of TuR from TrE. However, some limitations in this study should be noted. First, given the influence of reagents and patients' wishes, the sample size in this study is relatively small. Larger sample studies are needed to support our conclusions. Second, this cohort is biased to IDH1 mutated patients (24/37), and most patients were treated almost two years before this investigation. This finding may be due to the prevalence of IDH1 mutation in patients with relatively late recurrence, and IDH1 wild type patients are more likely to identify recurrence because of rapid disease progression. In addition, the high rate of IDH1 mutation on TrE in our data may be due to the radiosensitization and a less aggressive phenotype of IDH1 mutated gliomas [37]. Third, WHO tumour grade and other molecular profiles, such as 1p19q, may affect the process and metabolic level of glioma recurrence, which is also not further discussed in this study. Moreover, due to the lack of sufficient diagnostic gold standards at present, we can only use pathology combined with follow-up for final diagnosis, which is similar to that noted in many other studies [4, 16, 38, 39]. TrE also includes changes in different periods and TrE or recurrent tumours will often coexist. These tumours are difficult to distinguish in many cases. Finally, the L/G ratio reflects only a single voxel uptake of the lesion and does not include volume-based information; thus, this metric can yield false-negative results due to the obvious heterogeneity of glioma. This notion may be the reason why the sensitivity is relatively low compared with the specificity in this study.

## Conclusion

In conclusion, PET/CT is a powerful tool to distinguish glioma TuR from TrE, and ^18^F-FDOPA PET/CT exhibited remarkably improved differential diagnosis efficacy than ^13^N-NH_3_ and ^18^F-FDG. Moreover, considering that the combination of ^18^F-FDOPA and ^13^N-NH_3_ shows comparably diagnostic performance with ^18^F-FDOPA alone, ^18^F-FDOPA alone is a good choice for effective diagnosis and could facilitate more effective therapeutic decision-making for patients with suspected glioma recurrence. However, this conclusion should be confirmed in more studies with larger samples.

## Data Availability

The authenticity of this article has been validated by uploading the key raw data onto the Research Data Deposit public platform (www.researchdata.org.cn) with the approval RDD number RDDA2020001608.

## Abbreviations

CT: Computed tomography
MR: Magnetic resonance
TrE: Termed treatment effects
TuR: Tumour recurrence
PET/CT: Positron emission tomography/computed tomography
^18^F-FDG: ^18^F-fluorodeoxyglucose
^13^N-NH_3_: ^13^N-ammonia
^11^C-MET: ^11^C-methylmethionine
^18^F-FET: ^18^F-fluoroethyl-L-tyrosine
^18^F-FDOPA: ^18^F-fluoro-L-dihydroxy-phenylalanine
^18^F-FLT: ^18^F-fluorothymidine
^18^F-FMISO: ^18^F-fluoromisonidazole
WHO: World Health Organization
ROI: Region of interest
SUV: Standardized uptake value
L/G: Lesion-to-normal grey-matter cortex
CNGM: Contralateral normal grey-matter
RANO: Response Assessment in Neuro-Oncology
ROC: Receiver operating characteristic
BBB: Blood–brain barrier
LGG: Low-grade gliomas
